# Identification of significant gene biomarkers of low back pain caused by changes in the osmotic pressure of nucleus pulposus cells

**DOI:** 10.1038/s41598-020-60714-y

**Published:** 2020-02-28

**Authors:** Changsong Zhao, Xuemin Quan, Jie He, Rugang Zhao, Yao Zhang, Xin Li, Sheng Sun, Rui Ma, Qiang Zhang

**Affiliations:** Department of Orthopaedics, Beijing Ditan Hospital, Capital Medical University, 100015 Beijing, China

**Keywords:** Biomarkers, Molecular medicine

## Abstract

The incidence of intervertebral disc (IVD) degeneration disease, caused by changes in the osmotic pressure of nucleus pulposus (NP) cells, increases with age. In general, low back pain is associated with IVD degeneration. However, the mechanism and molecular target of low back pain have not been elucidated, and there are no data suggesting specific biomarkers of low back pain. Therefore, the research aims to identify and verify the significant gene biomarkers of low back pain. The differentially expressed genes (DEGs) were screened in the Gene Expression Omnibus (GEO) database, and the identification and analysis of significant gene biomarkers were also performed with various bioinformatics programs. A total of 120 patients with low back pain were recruited. Before surgery, the degree of pain was measured by the numeric rating scale (NRS), which enables comparison of the pain scores from individuals. After surgery, IVD tissues were obtained, and NP cells were isolated. The NP cells were cultured in two various osmotic media, including iso-osmotic media (293 mOsm/kg H_2_O) to account for the morbid environment of NP cells in IVD degeneration disease and hyper-osmotic media (450 mOsm/kg H_2_O) to account for the normal condition of NP cells in healthy individuals. The relative mRNA expression levels of CCL5, OPRL1, CXCL13, and SST were measured by quantitative real-time PCR in the *in vitro* analysis of the osmotic pressure experiments. Finally, correlation analysis and a neural network module were employed to explore the linkage between significant gene biomarkers and pain. A total of 371 DEGs were identified, including 128 downregulated genes and 243 upregulated genes. Furthermore, the four genes (CCL5, OPRL1, SST, and CXCL13) were identified as significant gene biomarkers of low back pain (P < 0.001) based on univariate linear regression, and CCL5 (odds ratio, 34.667; P = 0.003) and OPRL1 (odds ratio, 19.875; P < 0.001) were significantly related to low back pain through multivariate logistic regression. The expression of CCL5 and OPRL1 might be correlated with low back pain in patients with IVD degeneration disease caused by changes in the osmotic pressure of NP cells.

## Introduction

IVD is fibro-cartilage tissue located between vertebrae and is composed of the nucleus pulposus, annulus fibrosus and endplate^1^. NP survives in a special ecological niche containing no distribution of blood vessels and nerves in which the osmotic pressure is significantly higher than that of plasma, enabling proteoglycans to be present in high concentrations^2,3^. Increasing with age, the osmotic pressure changes, and the interverbal disc degenerates^4,5^, which may be related to low back pain^6,7^. Previous studies have shown that the osmotic environment can influence the gene expression of NP cells^7^; thus, we hypothesized that there are pain-related genes among these affected genes, which might provide new therapeutic strategies and drug targets for low back pain.

In addition, the NRS is ubiquitous in the clinic as a means of evaluating low back pain^8^. The scale requires patients to describe pain intensity using the numbers 0–10 or 0–100, while a higher number means more pain (0 = no pain, 100 or 100 = most severe pain)^9^. Therefore, the NRS was used to evaluate the degree of low back pain in this study.

Through bioinformatic analysis, we screened enrichment pathways, such as the regulation of extracellular matrix organization and the Janus kinase (JAK)/signal transducer and activator of transcription (STAT) signalling pathway, as well as key genes, such as CXCL13, SST, CCL5 and OPRL1, between NP cells cultured in hyperosmotic media and NP cells cultured in iso-osmotic media. In addition, through quantitative real-time PCR and statistical analysis, we found that CCL5 and OPRL1 might be potential biomarkers of low back pain.

## Results

### Identification of DEGs between NP cells cultured in hyperosmotic media and NP cells cultured in iso-osmotic media

**Figure 1:**
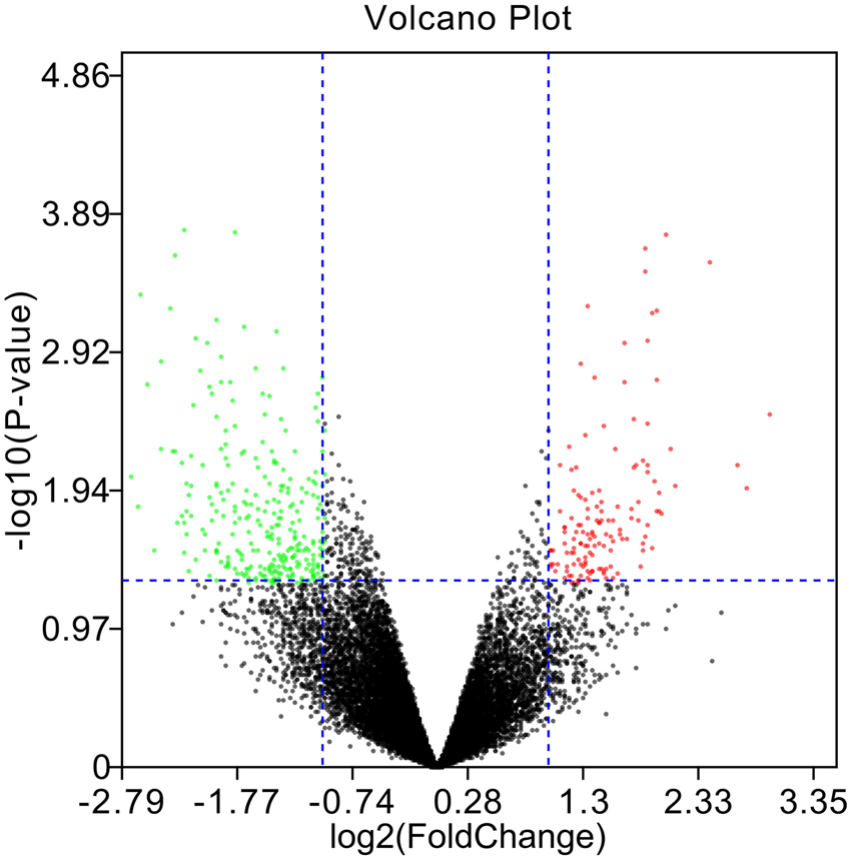
Volcano plot presents the DEGs between IVD cells cultured in hyperosmotic media and IVD cells cultured in iso-osmotic media. The downregulated DEGs are marked in green, and upregulated DEGs are marked in red.

After analysis of the datasets (GSE1648) with the limma package, the difference between NP cells cultured in hyperosmotic media and NP cells cultured in iso-osmotic media could be presented in a volcano plot. After setting the threshold value, a total of 371 DEGs were reserved, including 128 downregulated DEGs and 243 upregulated DEGs (Fig. 1).

### Functional annotation for DEGs via DAVID and Metascape

**Figure 2:**
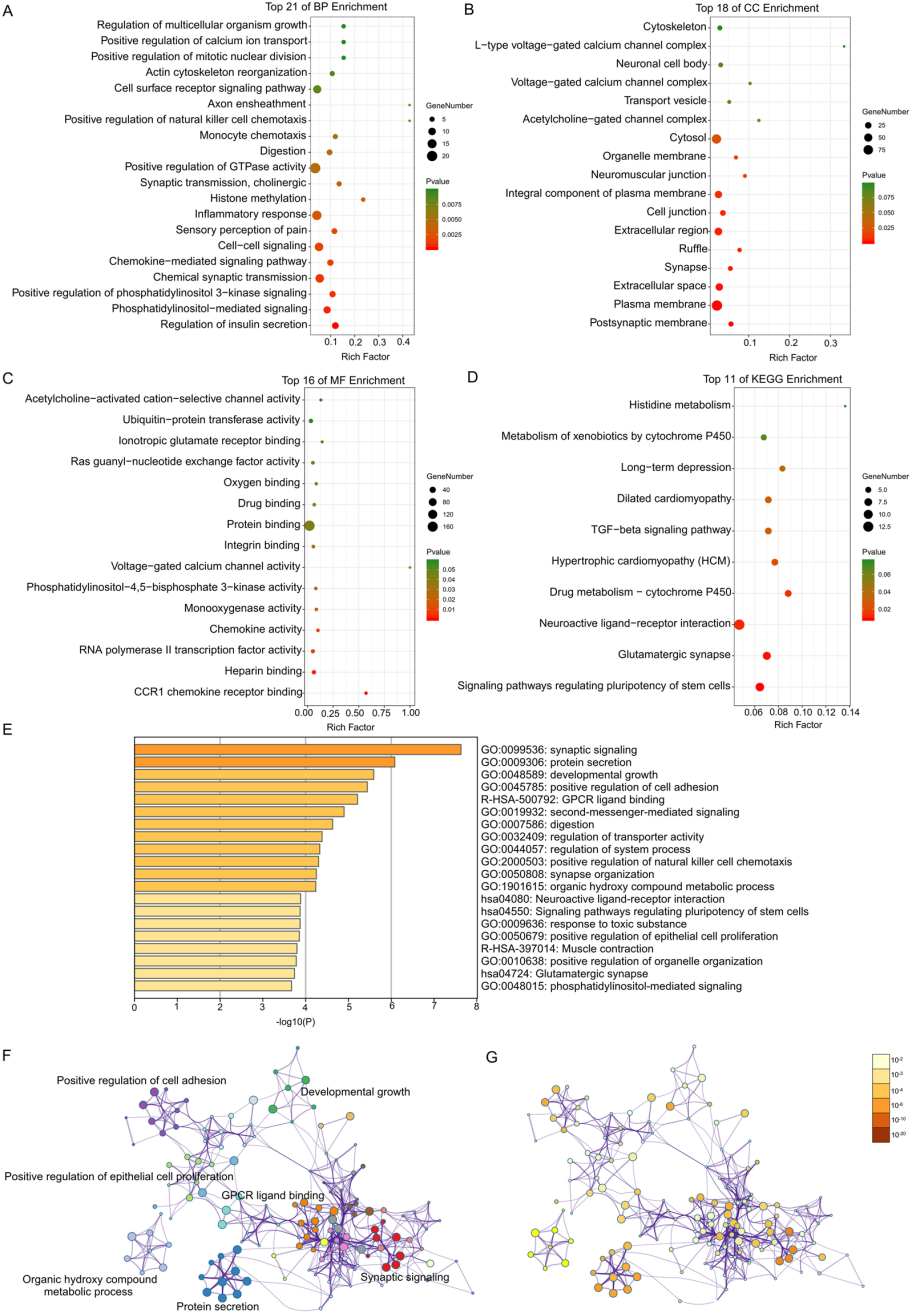
Enrichment analysis of DEGs by DAVID and Metascape. Detailed information relating to changes in the (**A**) cellular component, (**B**) biological process, (**C**) molecular function, and (**D**) KEGG analysis for hub genes. (**E**) Heatmap of enriched terms across input differentially expressed gene lists, coloured by p-values, via Metascape. (**F**) Network of enriched terms coloured by clust er identity, where nodes that share the same cluster identity are typically close to each other. (**G**) Network of enriched terms coloured by p-value, where terms containing more genes tend to have a more significant p-value.

Through DAVID analysis, the results of the Gene Ontology analysis showed that variations in DEGs linked with biological processes were mainly enriched in sensory perception of pain and inflammatory response (Fig. 2A). Variations in DEGs linked with cellular components were significantly enriched in the postsynaptic membrane, synapse, neuromuscular junction, acetylcholine−gated channel complex, voltage−gated calcium channel complex, and L−type voltage−gated calcium channel complex (Fig. 2B). With regard to molecular function, DEGs were significantly enriched in chemokine activity, monooxygenase activity, and voltage−gated calcium channel activity (Fig. 2C). Analysis of KEGG pathways indicated that the top canonical pathways associated with DEGs were glutamatergic synapses and neuroactive ligand−receptor interactions (Fig. 2D).

Furthermore, the functional enrichment analysis with Metascape showed that the DEGs between NP cells cultured in hyperosmotic media and NP cells cultured in iso-osmotic media were significantly enriched in synaptic signalling, synapse organization, neuroactive ligand-receptor interaction, and glutamatergic synapse (P < 0.05, Fig. 2E–G).

### Gene ontology and KEGG pathway enrichment analysis of DEGs in NP cells using GSEA

**Figure 3:**
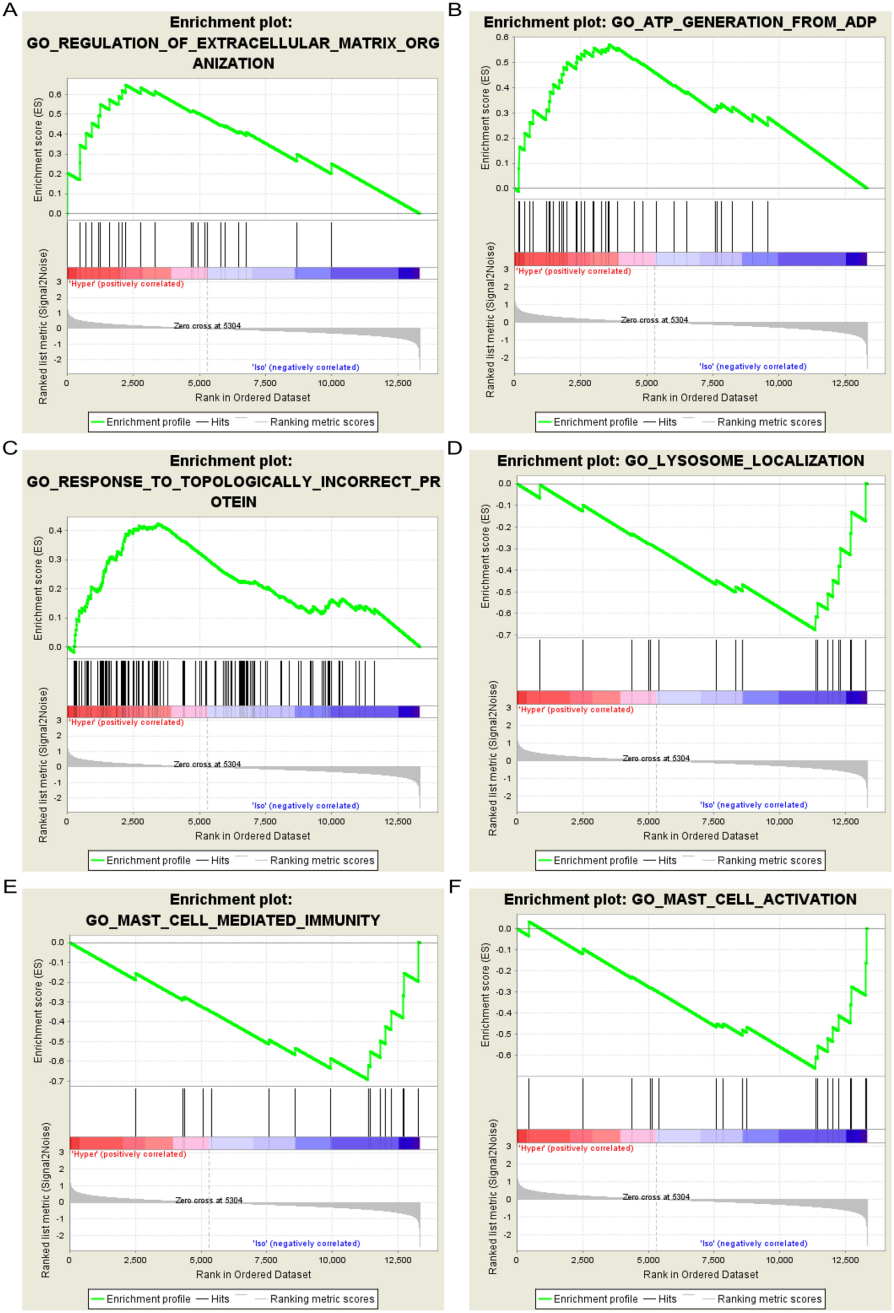
Six significant enrichment plots of functional enrichment analysis of DEGs between hyper and iso samples in GSE1648 using GSEA. (**A**) Enrichment plot: Gene Ontology_REGULATION_OF_EXTRACELLULAR_MATRIX_ORGANIZATION. Profile of the Running ES Score & Positions of GeneSet Members on the Rank Ordered List. (**B**) Enrichment plot: Gene Ontology_ATP_GENERATION_FROM_ADP. Profile of the Running ES Score & Positions of GeneSet Members on the Rank Ordered List. (**C**) Enrichment plot: Gene Ontology_RESPONSE_TO_TOPOLOGICALLY_INCORRECT_PROTEIN. Profile of the Running ES Score & Positions of GeneSet Members on the Rank Ordered List. (**D**) Enrichment plot: Gene Ontology_LYSOSOME_LOCALIZATION. Profile of the Running ES Score & Positions of GeneSet Members on the Rank Ordered List. (**E**) Enrichment plot: Gene Ontology_MAST_CELL_MEDIATED_IMMUNITY. Profile of the Running ES Score & Positions of GeneSet Members on the Rank Ordered List. (**F**) Enrichment plot: Gene Ontology_MAST_CELL_ACTIVATION. Profile of the Running ES Score & Positions of GeneSet Members on the Rank Ordered List.

**Figure 4:**
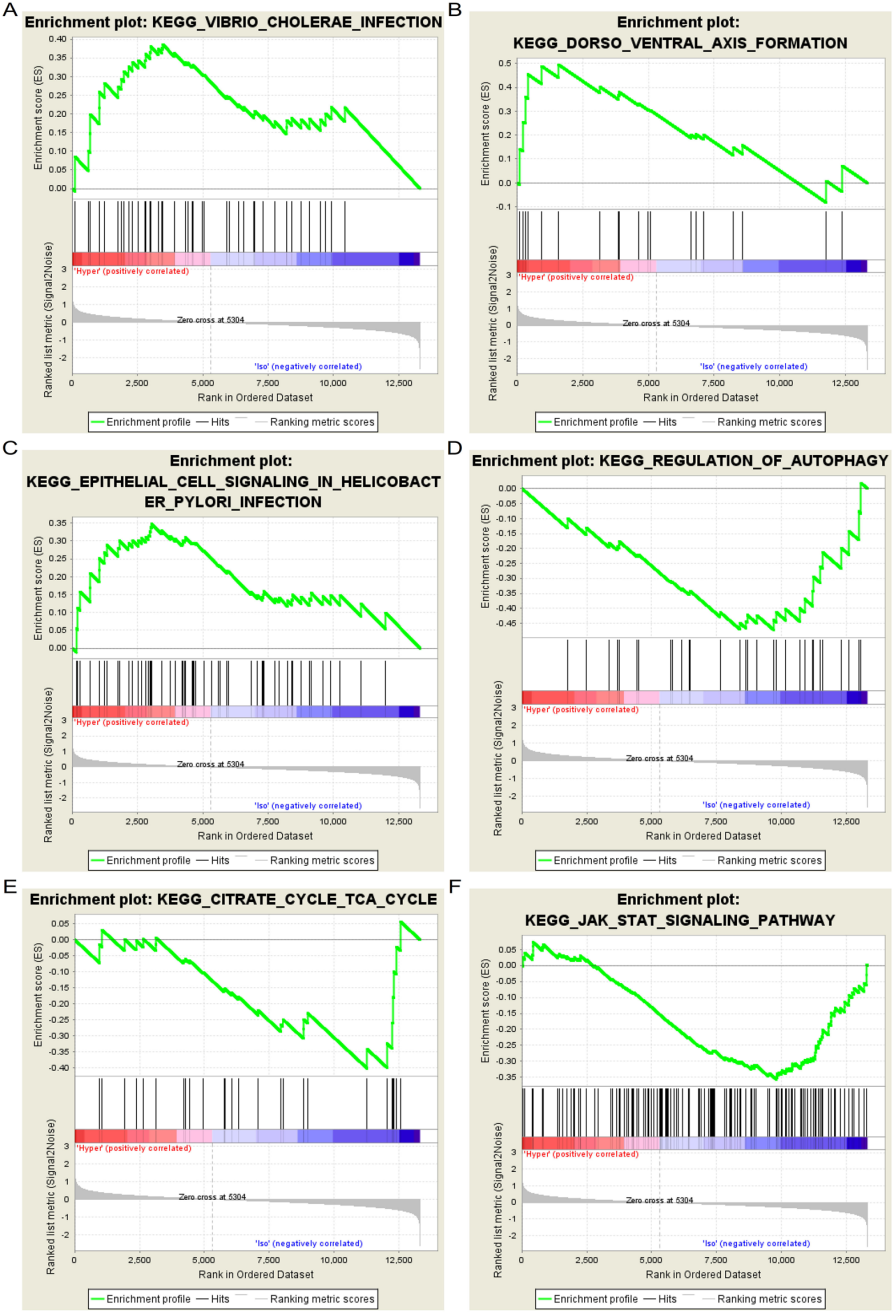
Six significant enrichment plots of pathway enrichment analysis of DEGs between hyper and iso samples in GSE1648 using GSEA. (**A**) Enrichment plot: KEGG_VIBRIO_CHOLERAE_INFECTION. Profile of the Running ES Score & Positions of GeneSet Members on the Rank Ordered List. (**B**) Enrichment plot: KEGG_DORSO_VENTRAL_AXIS_FORMATION. Profile of the Running ES Score & Positions of GeneSet Members on the Rank Ordered List. (**C**) Enrichment plot: KEGG_EPITHELIAL_CELL_SIGNALING_IN_HELICOBACTER_PYLORI_INFECTION. Profile of the Running ES Score & Positions of GeneSet Members on the Rank Ordered List. (**D**) Enrichment plot: KEGG_REGULATION_OF_AUTOPHAGY. Profile of the Running ES Score & Positions of GeneSet Members on the Rank Ordered List. (**E**) Enrichment plot: KEGG_CITRATE_CYCLE_TCA_CYCLE. Profile of the Running ES Score & Positions of GeneSet Members on the Rank Ordered List. (**F**) Enrichment plot: KEGG_JAK_STAT_SIGNALING_PATHWAY. Profile of the Running ES Score & Positions of GeneSet Members on the Rank Ordered List.

The results of Gene Ontology enrichment analysis by GSEA indicated that 1274/4081 genes were upregulated in hypertonic NP cells. Table 1 lists the most significant enrichments in the upregulated and downregulated gene sets according to the normalized enrichment score by order (Table 1). Figure 3 shows the six most significant plots in the upregulated and downregulated gene sets. KEGG enrichment analysis by GSEA indicated that 70/168 gene sets were upregulated in hypertonic NP cells compared to isotonic NP cells, while 98/168 gene sets were downregulated. Table 2 lists the most significant enrichments in the upregulated and downregulated gene sets according to the normalized enrichment score by order (Table 2). Figure 4 shows the six most significant plots in the upregulated and downregulated gene sets.

**Table 2:** Pathway enrichment analysis of DEGs in IVD cells using GSEA.

Gene Set Name	SIZE	ES	NES	p-val
**Upregulated**				
KEGG_VIBRIO_CHOLERAE_INFECTION	46	0.386	1.418	0.068
KEGG_DORSO_VENTRAL_AXIS_FORMATION	19	0.494	1.375	0.128
KEGG_EPITHELIAL_CELL_SIGNALING_IN_HELICOBACTER_PYLORI_INFECTION	58	0.348	1.338	0.025
KEGG_ERBB_SIGNALING_PATHWAY	82	0.325	1.323	0.019
KEGG_FRUCTOSE_AND_MANNOSE_METABOLISM	32	0.388	1.312	0.058
KEGG_NEUROTROPHIN_SIGNALING_PATHWAY	117	0.312	1.312	0.048
**Downregulated**				
KEGG_REGULATION_OF_AUTOPHAGY	31	0.472	1.752	0.000
KEGG_CITRATE_CYCLE_TCA_CYCLE	29	0.401	1.515	0.000
KEGG_JAK_STAT_SIGNALING_PATHWAY	140	0.358	1.456	0.059
KEGG_MISMATCH_REPAIR	22	0.445	1.445	0.060
KEGG_HOMOLOGOUS_RECOMBINATION	25	0.458	1.416	0.053
KEGG_CYTOSOLIC_DNA_SENSING_PATHWAY	47	0.464	1.394	0.086

IVD: Intervertebral disc; ES: Enrichment Score; NES: Normalized Enrichment Score.

**Table 1:** Functional enrichment analysis of DEGs in IVD cells using GSEA.

Gene Set Name	SIZE	ES	NES	P-value
**Upregulated**				
REGULATION_OF_EXTRACELLULAR_MATRIX_ORGANIZATION	25	0.647	2.294	0.000
ATP_GENERATION_FROM_ADP	35	0.571	2.175	0.000
RESPONSE_TO_TOPOLOGICALLY_INCORRECT_PROTEIN	134	0.425	2.127	0.000
RIBONUCLEOSIDE_DIPHOSPHATE_METABOLIC_PROCESS	54	0.495	2.121	0.000
ADP_METABOLIC_PROCESS	41	0.519	2.098	0.000
GLUCOSE_CATABOLIC_PROCESS	28	0.574	2.060	0.000
**Downregulated**				
LYSOSOME_LOCALIZATION	18	0.676	1.855	0.000
MAST_CELL_MEDIATED_IMMUNITY	16	0.693	1.842	0.004
MAST_CELL_ACTIVATION	19	0.662	1.810	0.003
NEGATIVE_REGULATION_OF_LIPID_CATABOLIC_PROCESS	17	0.677	1.802	0.001
RNA_PHOSPHODIESTER_BOND_HYDROLYSIS_ENDONUCLEOLYTIC	41	0.559	1.782	0.001
NEUROPEPTIDE_RECEPTOR_ACTIVITY	30	0.584	1.773	0.000

IVD: Intervertebral disc; ES: Enrichment Score; NES: Normalized Enrichment Score.

**Table 3:** Summaries for the function of 4 significant genes.

No.	Gene symbol	Full name	Function
1	CCL5	C-C Motif Chemokine Ligand 5	Chemoattractant for blood monocytes, memory T-helper cells and eosinophils. Causes the release of histamine from basophils and activates eosinophils.
2	OPRL1	Opioid Related Nociceptin Receptor 1	G-protein coupled opioid receptor that functions as receptor for the endogenous neuropeptide nociceptin.
3	SST	Somatostatin	Somatostatin (somatotropin release inhibiting factor, SRIF) is an endogenous cyclic polypeptide with two biologically active forms. It is an abundant neuropeptide and has a wide range of physiological effects on neurotransmission, secretion and cell proliferation.
4	CXCL13	C-X-C Motif Chemokine Ligand 13	Chemotactic for B-lymphocytes but not for T-lymphocytes, monocytes and neutrophils. Does not induce calcium release in B-lymphocytes.

### PPI construction and module analysis

**Figure 5:**
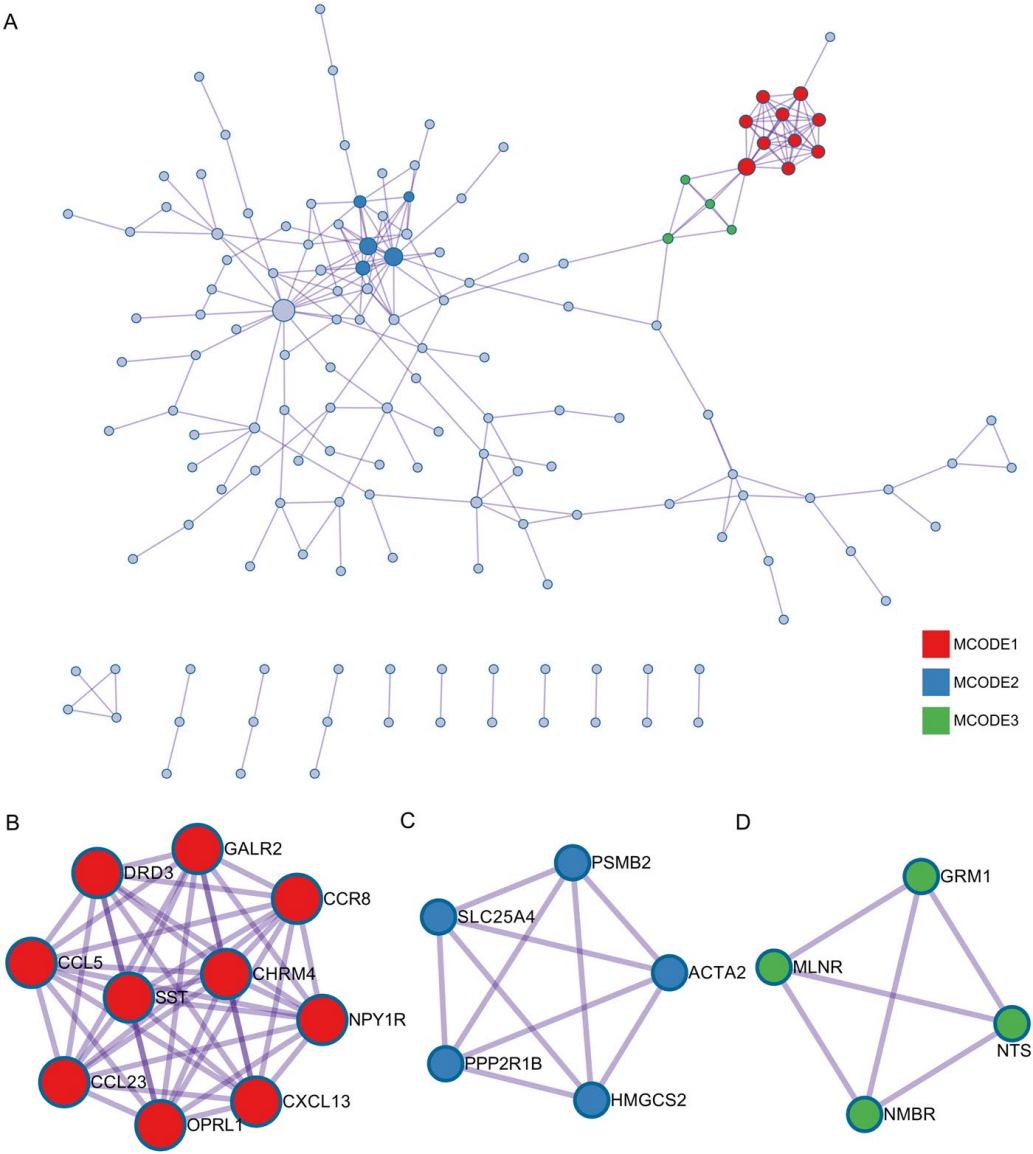
PPI network and significant module constructed by Metascape. (**A**) Through Metascape analysis, a protein-protein interaction network of the DEGs was constructed. (**B**) MCODE1 consists of 10 genes, including GALR2, CCR8, NPY1R, CXCL13, OPRL1, CCL23, CCL5, DRD3, SST, and CHRM4. (**C**) MCODE2 consists of 5 genes, including PSMB2, ACTA2, SLC25A4, PPP2R1B, and HMGCS2. (**D**) MCODE3 consists of 4 genes, including GRM1, NTS, NMBR, and MLNR.

Through Metascape analysis, a protein-protein interaction network of the DEGs was constructed (Fig. 5A). Three MCODE modules were identified from the PPI network. First, MCODE1 consisted of 10 genes, including GALR2, CCR8, NPY1R, CXCL13, OPRL1, CCL23, CCL5, DRD3, SST, and CHRM4 (Fig. 5B). Second, MCODE2 consisted of 5 genes, including PSMB2, ACTA2, SLC25A4, PPP2R1B, and HMGCS2 (Fig. 5C). Third, MCODE3 consisted of 4 genes, including GRM1, NTS, NMBR, and MLNR (Fig. 5D).

**Figure 6:**
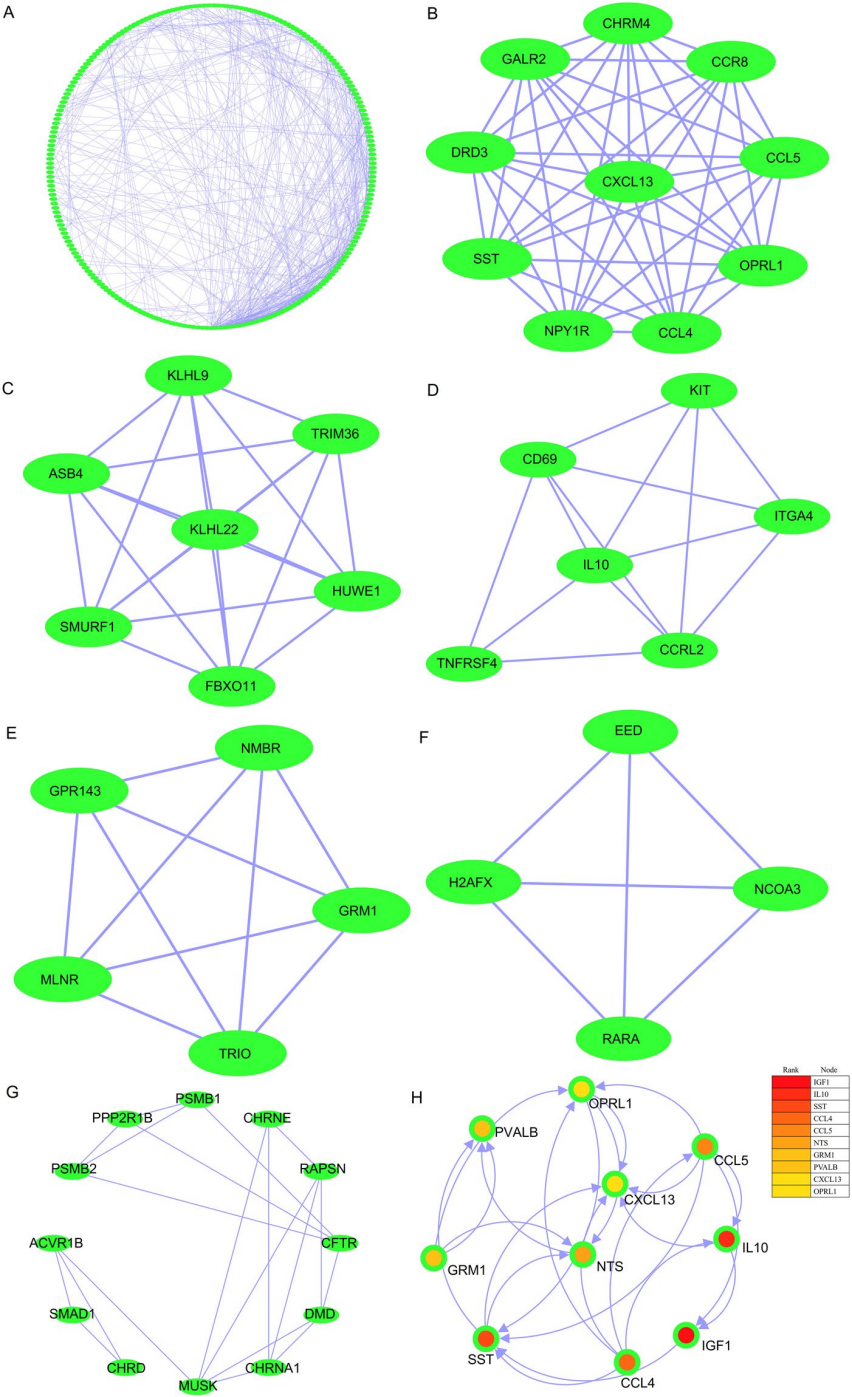
PPI network, significant module, and hub gene network constructed by STRING and Cytoscape. (**A**) There were 539 edges and 225 nodes in the PPI network. (**B**) The first module consists of 45 edges and 10 nodes. (**C**) The second module consists of 21 edges and 7 nodes. (**D**) The third module consists of 13 edges and 6 nodes. (**E**) The fourth module consists of 10 edges and 5 nodes. (**F**) The fifth module consists of 6 edges and 4 nodes. (**G**) The sixth module consists of 20 edges and 12 nodes. (**H**) A total of 10 genes were identified as hub genes with Cytoscape: OPRL1, CCL5, IL10, IGF1, CCL4, SST, GRM1, PVALB, CXCL13, and NTS.

Furthermore, construction of the PPI network was also performed by the STRING tool, and there were 539 edges and 225 nodes in the PPI network (PPI enrichment p-value < 0.05) (Fig. 6A). Six MCODE modules were also identified from the PPI network by the Molecular Complex Detection tool (MCODE) (version 1.5.1), one plug-in of Cytoscape (Fig. 6B–G). Degrees >10 were considered the criterion of judgement. A total of 10 genes were identified as hub genes with Cytoscape: OPRL1, CCL5, IL10, IGF1, CCL4, SST, GRM1, PVALB, CXCL13, and NTS (Fig. 6H).

### Identification and analysis of significant genes

**Figure 7:**
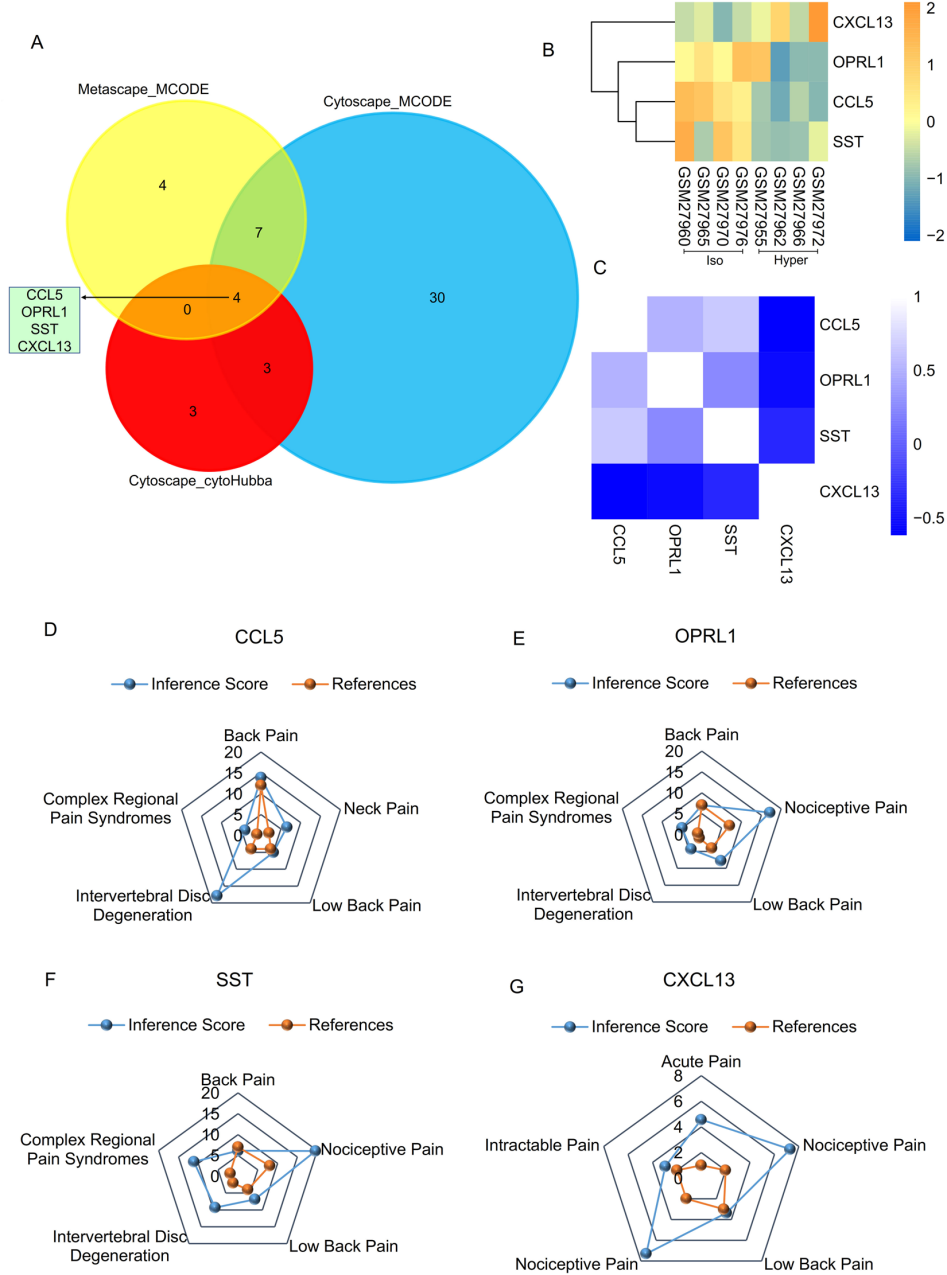
Identification and analysis of significant genes. (**A**) The VENN diagram shows that there were four significant common genes among “Metascape_MCODE”, “Cytoscape_MCODE”, and “Cytoscape_cytoHubba”, including CCL5, OPRL1, SST, and CXCL13. (**B**) Hierarchical clustering showed that the significant genes could basically differentiate the IVD cells cultured in hyperosmotic media from the IVD cells cultured in iso-osmotic media. (**C**) The Pearson correlation analysis showed positive correlations among CCL5, OPRL1, and SST. However, the expression of CXCL13 was negatively related to the expression of CCL5, OPRL1, and SST. (**D**) Relationship to pain and intervertebral disc degeneration related to CCL5 based on the CTD database. (**E**) Relationship to pain and intervertebral disc degeneration related to OPRL1 based on the CTD database. (**F**) Relationship to pain and intervertebral disc degeneration related to SST based on the CTD database. (**G**) Relationship to pain and intervertebral disc degeneration related to CXCL13 based on the CTD database.

The VENN diagram showed that there were four significant common genes among “Metascape_MCODE”, “Cytoscape_MCODE”, and “Cytoscape_cytoHubba”, including CCL5, OPRL1, SST, and CXCL13 (Fig. 7A). Summaries of the functions of the four significant genes are shown in Table 3. Hierarchical clustering showed that the significant genes could largely differentiate the NP cells cultured in hyperosmotic media from the NP cells cultured in iso-osmotic media. Compared with NP cells cultured in hyperosmotic media, the expression of CXCL13 was downregulated, and the expression of OPRL1, CCL5, and SST was upregulated, in NP cells cultured in iso-osmotic media (Fig. 7B). The Pearson correlation analysis showed that there was a positive correlation between CCL5 and OPRL1 (Fig. 7C). Identification of significant genes associated with pain and intervertebral disc degeneration was performed on the comparative toxicogenomics database, which is shown in Fig. 7D–G.

### Results of quantitative real-time PCR analysis

**Figure 8:**
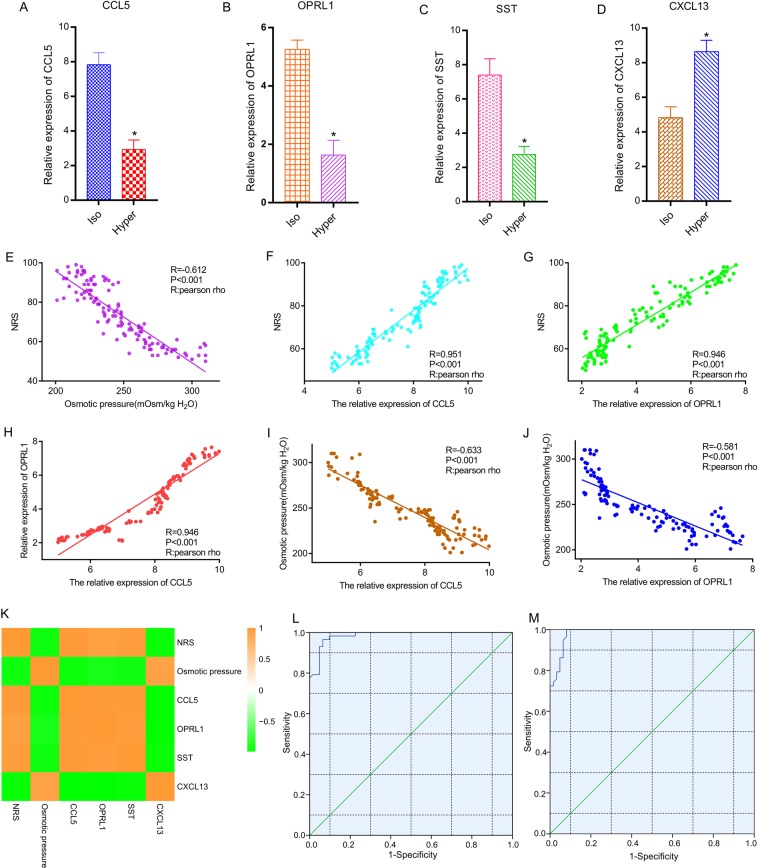
Expression level of significant genes through quantitative real-time PCR analysis and strong associations between NRS, osmotic pressure, CCL5, and OPRL1. (**A**) The relative expression level of CCL5. (**B**) The relative expression level of OPRL1. (**C**) The relative expression level of SST. (**D**) The relative expression level of CXCL13. (**E**) The NRS was negatively associated with osmotic pressure. (**F**) There is a positive association between NRS and the relative expression of CCL5. (**G**) The NRS was positively associated with the relative expression of OPRL1. (**H**) There was also a positive association between the relative expression of OPRL1 and the relative expression of CCL5. (**I**) Osmotic pressure was negatively associated with the relative expression of CCL5. (**J**) Osmotic pressure was negatively associated with the relative expression of OPRL1. (**K**) The heatmap shows the strong associations between the NRS, osmotic pressure, CCL5, OPRL1, SST, and CXCL13 through Spearman correlation analysis. (**L**) The receiver operator characteristic curve indicates that the expression level of CCL5 could predict NRS sensitively and specifically. (**M**) The receiver operator characteristic curve indicates that the expression level of OPRL1 could also predict NRS sensitively and specifically.

The relative expression levels of CCL5 (Fig. 8A), OPRL1 (Fig. 8B), and SST (Fig. 8C) were significantly higher in the NP cells cultured in iso-osmotic media than in the NP cells cultured in hyper-osmotic media. However, the relative expression levels of CXCL13 were reversed (Fig. 8D).

### miRNA of the four significant genes prediction

The miRNAs that regulate the four significant genes were screened out with TargetScan (Table 4).

**Table 4:** Summary of miRNAs that regulate hub genes.

	Gene	Predicted MiR
1	CCL5	hsa-miR-5000-5p hsa-miR-148a-5p hsa-miR-5010-3p
2	OPRL1	hsa-miR-31-5p
3	SST	hsa-miR-101-3p.2
4	CXCL13	hsa-miR-2116-5p hsa-miR-4677-5p hsa-miR-22-5p

### Strong associations between the NRS, osmotic pressure, CCL5, and OPRL1

The NRS was negatively associated with osmotic pressure (Pearson Rho = −0.612, P < 0.001) (Fig. 8E). The NRS was positively associated with the relative expression of CCL5 (Pearson Rho = 0.951, P < 0.001) (Fig. 8F) and OPRL1 (Pearson Rho = 0.946, P < 0.001) (Fig. 8G). There was also a positive association between OPRL1 and CCL5 (Pearson Rho = 0.946, P < 0.001) (Fig. 8H). Osmotic pressure was negatively associated with CCL5 (Pearson Rho = −0.633, P < 0.001) (Fig. 8I) and OPRL1 (Pearson Rho = −0.581, P < 0.001) (Fig. 8J). The heatmap showed strong associations between the NRS, osmotic pressure, CCL5, OPRL1, SST, and CXCL13. There was a positive correlation between CCL5 and OPRL1 (Fig. 8K).

### Further associations between NRS and relevant gene expression based on univariate and multiple linear regression

Univariate linear regression showed that the NRS was significantly correlated with CCL5 (β = 0.951, p < 0.001), OPRL1 (β = 0.946, p < 0.001), SST (β = 0.902, p < 0.001), and CXCL13 (β = −0.958, p < 0.001). Furthermore, the NRS remained related to CCL5 (β = 0.273, p = 0.024), OPRL1 (β = 0.224, p = 0.036), and CXCL13 (β = −0.389, p = 0.007) in the multivariate linear regression model (Table 5).

**Table 5:** Linear regression analysis between NRS and relevant gene expression.

Gene symbol	NRS			
	Univariate linear regression		Multiple linear regression	
	β^a^	P-value	β^b^	P-value
CCL5	0.951	<0.001*	0.273	0.024*
OPRL1	0.946	<0.001*	0.224	0.036*
SST	0.902	<0.001*	0.094	0.167
CXCL13	−0.958	<0.001*	−0.389	0.007*

^a^Univariate linear regression; ^b^Multiple linear regression analysis; β: parameter estimate; NRS: numeric rating scale.

*Significant variables: P<0.05.

### Univariate logistic regression for the proportional hazard analysis of relevant gene expression for NRS

Table 6 presents the univariate odds ratio (OR) and 95% confidence intervals (95% CI) for the NRS of significant genes. The OR for NRS was 296.400 (95% CI, 36.672–2395.665, p < 0.001) in the group with high expression of CCL5 compared with the low-expression group. For the NRS, subjects with high expression of OPRL1 had a higher OR of 153.700 (95% CI, 39.193–602.760, p < 0.001) than subjects with low expression. However, there was no significant detrimental impact of SST and CXCL13 on the NRS (Table 6).

**Table 6:** Correlative parameters’ effect on NRS based on univariate logistic proportional regression analysis.

Parameters		NRS		
		OR	95% CI	P
CCL5	Low	1		<0.001*
	High	296.400	36.672–2395.665	
OPRL1	Low	1		<0.001*
	High	153.700	39.193–602.760	
SST	Low	1		0.997
	High	9.1×10^9^	0.000–0.000	
CXCL13	Low	1		0.996
	High	0.000	0.000–0.000	

OR, odds ratio; 95% CI, 95% confidence interval; NRS: numeric rating scale. *P < 0.05.

### Independent risk factors for NRS based on multivariate logistic regression

The result of multivariate logistic proportional regression analysis showed that higher expression of CCL5 in individuals was associated with significantly greater risk, and the OR of high CCL5 was 34.667 (95% CI, 3.311–362.993; p = 0.003). Additionally, the OR of high OPRL1 was 19.875 (95% CI, 3.922–100.711; p < 0.001) (Table 7).

**Table 7:** Correlative genes’ effect on NRS based on multiple logistic proportional regression analysis.

Genes	NRS		
	OR	95% CI	P
CCL5	34.667	3.311–362.993	0.003*
OPRL1	19.875	3.922–100.711	<0.001*

NRS: numeric rating scale; OR, odds ratio; 95% CI, 95% confidence interval. *P < 0.05.

### Expression of CCL5 and OPRL1 can sensitively and specifically predict NRS through the receiver operating characteristic curve

The receiver operator characteristic curve indicated that the expression level of CCL5 could predict NRS sensitively and specifically (area under the curve for NRS, 0.985; p < 0.001) (Fig. 8L), and the expression level of OPRL1 could also predict NRS sensitively and specifically (area under the curve for NRS, 0.985; p < 0.001) (Fig. 8M) (Table 8).

**Table 8:** Receiver operator characteristic curve analysis of relative gene expression for NRS.

Gene symbol	NRS			
	AUC	P-value	95% CI	ODT
CCL5	0.985	<0.001*	0.970–1.000	8.035
OPRL1	0.985	<0.001*	0.970–1.000	3.840

AUC: area under curve; max the maximum of AUC; *Significant variables; ODT: Optimal diagnostic threshold; NRS: numeric rating scale.

### Neural network prediction model and high-risk warning range of NRS

**Figure 9:**
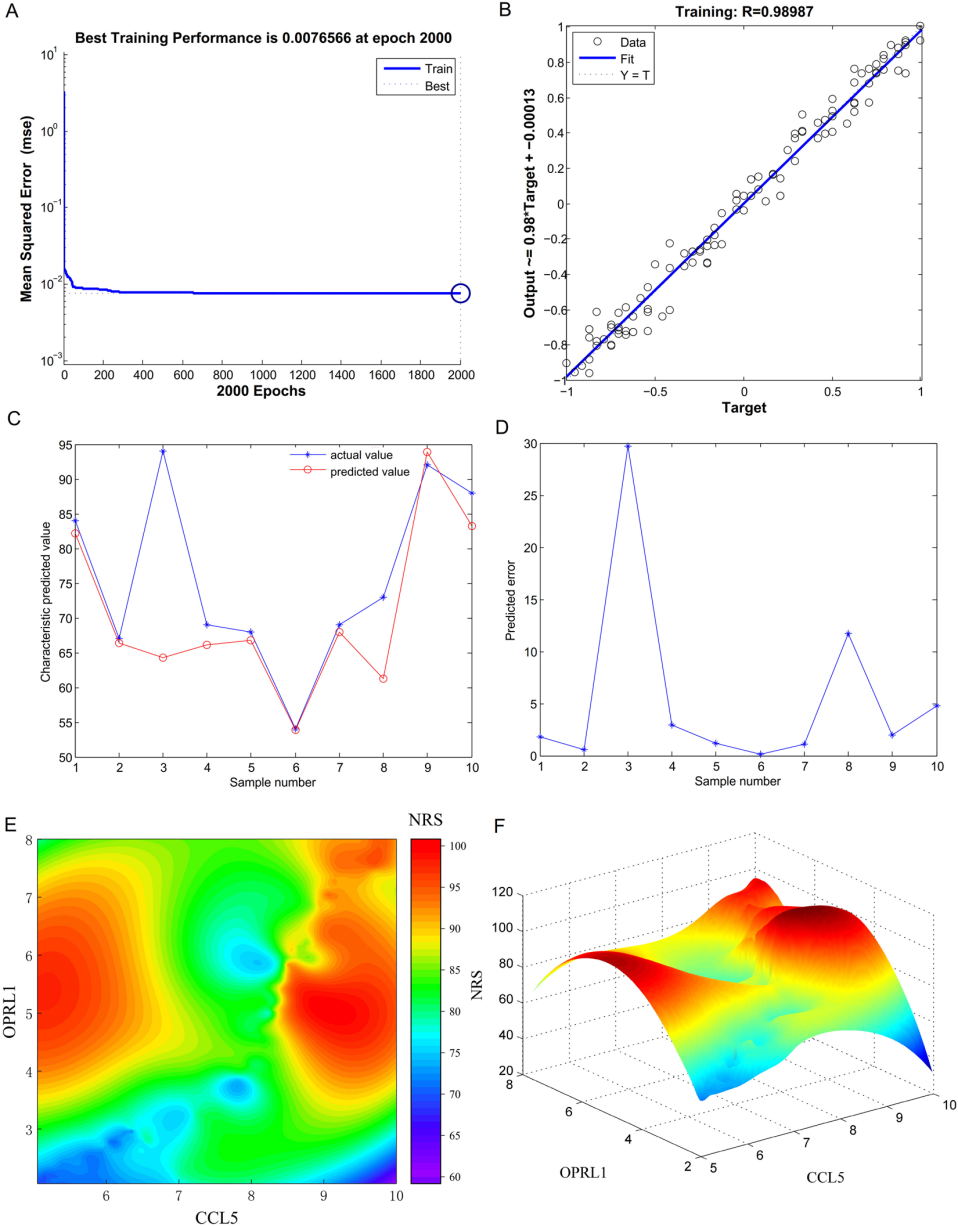
Neural network prediction model and high-risk warning range of NRS. (**A**) The neural network prediction model reached the best effect, in which the mean squared error was 0.0076566 at epoch 2000. (**B**) The final training model of the neural network prediction model, and the relativity is 0.98987. (**C**) Verify the predicted value of the data against the actual value. (**D**) Verify the data error percentage curve. (**E**) The high-risk warning range of NRS at the level of the planform. (**F**) The high-risk warning range of NRS at the level of the three-dimensional stereogram.

After training, the neural network prediction model reached the best effect, in which the mean squared error was 0.0076566 at epoch 2000 (Fig. 9A), and the relativity was 0.98987 (Fig. 9B). By verifying the predicted value of the data against the actual value, we found that there are only small differences (Fig. 9C,D).

Through the cubic spline interpolation algorithm, we found the high-risk warning indicator of NRS: CCL5 <7 or> 8.5, and 3.5 <OPRL1 <8 (Fig. 9E). Furthermore, the three-dimensional stereogram could accurately present the warning range (Fig. 9F).

## Discussion

Low back pain is a common symptom experienced by people of all ages. The median prevalence rate in high-income countries is as high as 30.3%, which considerably increases the economic burden^10^. The aetiology and mechanism of low back pain are highly complicated. Previous studies showed that disc degeneration is closely related to low back pain^11^. The intervertebral disc is in a special hypertonic environment^2^, and its degeneration includes changes in the number of intervertebral disc cells and gene expression, as well as changes in extracellular matrix synthesis and catabolism containing changes in osmotic pressure^4,5^. The aim of this study was to explore the effects of intervertebral disc osmotic pressure on intervertebral disc cells from gene expression levels to identify potential therapeutic targets and disease-associated genes.

Biological information analysis has been widely applied to explore gene changes in the process of disease development, and this method may help scientists to identify new therapeutic targets^12–14^. A total of 371 DEGs, including 243 upregulated and 128 downregulated DEGs, were screened between NP cells cultured in hyperosmotic media and NP cells cultured in iso-osmotic media by analysing the biological data of the gene expression profile GSE1648. Through gene enrichment analysis of DEGs, the influence of osmotic pressure on gene expression in NP cells was comprehensively summarized: regulation of extracellular matrix organization and the JAK/STAT signalling pathway.

The extracellular matrix is mainly composed of water (60–99% by weight), collagen and proteoglycan^15^. Changes in osmotic pressure could affect the expression of some molecules in the extracellular matrix, and a large number of previous experiments demonstrated this phenomenon^7,16–19^. Ishihara used the NP of bovine tailbone for culture and found that the rate of proteoglycan synthesis was significantly increased when the osmotic pressure was 430 mOsm compared to 280 mOsm^17^. Takeno used bovine NP cells for *in vitro* culture and found that glycosaminoglycan products were in the highest concentration at 370 mOsm and in the lowest concentration at 270 mOsm^19^. Neidlinger-wilke obtained similar results by using bovine NP. When osmotic pressure increased from 300 mOsm to 500 mOsm, the expression of proteoglycan and matrix metallopeptidase-2 was upregulated, while the expression of matrix metallopeptidase-3 was downregulated^18^. However, Haschtmann found that different osmotic pressures had no significant effect on proteoglycan, but collagen I expression was upregulated when osmotic pressure was increased (485 mOsm compared to 335 mOsm)^16^. In addition to animal experiments, Wuertz found that collagen II was upregulated while collagen I was downregulated when the osmotic pressure was increased (300 mOsm compared to 500 mOsm) by using human NP cells^7^. The above evidence indicated that proteoglycan synthesis increased under hypertonic conditions. Some studies pointed out that the decrease in proteoglycan could damage the function of interverbal discs, aggravate disc degeneration, and promote angiogenesis^3,20^. Therefore, we believed that a decrease in osmotic pressure affected the synthesis of extracellular matrix and thus produced a negative impact on the disc. Artificial regulation of osmotic pressure under pathological conditions could possibly improve the function of the disc indirectly, thereby achieving pain relief and treatment. Although the molecular mechanism of osmotic pressure affecting the extracellular matrix has not been determined, some scholars have proposed that Tonicity-responsive enhancer binding protein (TonEBP) might play an important role in this process^21^. The survival of intervertebral disc cells in hypertonic media was closely related to the activation of TonEBP. TonEBP not only activates a series of gene products to make NP cells adapt to changes in the microenvironment but also plays an active role in protecting nucleus pulpotomy from apoptosis and regulating extracellular matrix components^22^. TonEBP is involved in inducing the expression of beta 1,3-glucuronosyl transferase 1, which is a key enzyme for glycosaminoglycan synthesis^22,23^.

The JAK/STAT signalling pathway, as an important pathway of intracellular signal transduction, is a downstream mediator of a variety of cytokines, hormones and growth factors^24^. When ligands bind to receptors, JAKs are activated, and the activated JAKs self-phosphorylate and phosphorylate the receptors. JAK-mediated phosphorylation activated STAT and thus led to the formation of dimers that directly bind to DNA and regulate gene expression^25^. Gabr showed that the expression of interleukin-17 increased in lumbar disc herniation and degeneration^26^, while Hu found that interleukin-17 upregulated the expression of VEGF in rat nucleus myeloid cells through the JAK/STAT pathway^27^. Chen proposed that interleukin-21 played a role in the pathological process of disc degeneration and could aggravate disc degeneration by stimulating TNF-α through the JAK/STAT pathway^28^. The study of Osuka showed that the JAK/STAT pathway was activated in lumbar disc herniation and played a role in inflammation, while the activation of the JAK/STAT pathway might be related to interleukin-6^29^. In conclusion, the expression of the JAK/STAT pathway might adversely affect the intervertebral disc, but few relevant studies could be retrieved at present; this topic might be one of our future research directions.

In addition, we found that there was a positive association between NRS and the relative expression of CCL5 (Pearson Rho = 0.951, P < 0.001) in low back pain, as well as OPRL1 (Pearson Rho = 0.946, P < 0.001), and CCL5 <7 or> 8.5 and 3.5 <OPRL1 <8 were high-risk warning indicators of NRS. These findings might assist us in evaluating patients’ pain more objectively and provide timely medical assistance, but its clinical applicability remains to be discussed.

CCL5, a protein-coding gene also known as RANTES (reduced upon^30^ activation of normal T cells expressed and secreted), functions as a chemoattractant for blood mononuclear cells, memory T cells and eosinophils, enables basophils to release histamine and activate eosinophils and not only plays an important role in the inflammatory response^31^ but also has a non-negligible influence on different pathological pain processes, which has been described in the literature. Oh found that subcutaneous injection of CCL5 in mice produced allodynia^32^. Pevida found that intraplantar injection of CCL5 caused thermal hyperalgesia in mice, which might be induced by activation of CCR1 or CCR5^33^. Kepler noted that CCL5 was significantly higher in human-derived painful NP cells than in painless discs^34^. Liou demonstrated that the lack of CCL5 reduced the recruitment of inflammatory cells to painful and inflamed sites and reduced pain in a mouse model of chronic neuropathic pain^35^. In addition, the analgesic activity of morphine could be attenuated by chemokines, especially CCL5 and CXCL12^36^. The above evidence suggested that the expression of CCL5 was associated with pain production or hyperalgesia. Phillips reported that CCL5 was upregulated in degenerate intervertebral disc samples compared to nondegenerated samples, but it was greater in infiltrated samples compared to degenerated samples. In addition, the team also presented the expression patterns of other cytokines and chemokines between different samples, indicating that it was necessary to investigate the relationship between the cytokine and chemokine and intervertebral disc degeneration^37^. CCL5 and other cytokines and chemokines are produced and secreted by intervertebral disc cells, and its release is increased in response to degenerative conditions^38^. CCL5 could promote matrix degradation, changes in cell phenotype, and infiltration and activation of inflammatory cells, further amplifying the inflammatory cascade, which is attributed to degeneration^39^. In the present study, the expression of CCL5 in isotonic samples was higher than that in hypertonic samples, indicating that changes in osmotic pressure affected the expression of pain-related factors in intervertebral disc cells, which were involved in the low back pain that occurred under pathological conditions and might become a new target for treating related diseases.

The protein encoded by OPRL1 is a member of the 7 transmembrane G protein-coupled receptor family, which acts as a receptor for endogenous nociceptin/orphanin FQ and is involved in a variety of biological functions and the regulation of neurobehavioral behaviours, including depression, anxiety, learning, memory, motor activity and drug dependence and addiction^40–43^. The effects of OPRL1 on modulating pain are bidirectional and can cause pain and analgesia^44^. Dagnino found that the OPRL1 antagonist UFP-101 exerted an analgesic effect in a mouse model of fibromyalgia induced by reserpine^45^, and similar findings had also been reported by^46^ Calo and Rizzi^47^. Zhang found that intraperitoneal injection of the OPRL1 antagonist JTC-801 reversed pain and anxiety caused by post-traumatic stress disorder (PTSD) in mice^48^. However, Andero pointed out that the OPRL1 agonist SR-8993 could prevent the accumulation of fear memory in the amygdala in a mouse model of PTSD and thus might prevent the occurrence of PTSD^49^. Ko found that subcutaneous injection of the OPRL1 agonist Ro64–6198 in monkeys produced antinociceptive effects against acute noxious stimuli and capsaicin-induced allodynia^50^. Rutten found that the OPRL1 agonist Ro65–6570 had an analgesic effect in diabetic mice^51^. In addition, changes in the expression of OPRL1 have also been reported in the literature. OPRL1 gene expression was increased after sciatic nerve ligation in mice^52^, and OPRL1 was upregulated on the dorsal root ganglion (DRG) in mice with neuropathic and inflammatory pain^53^. Anand found that OPRL1-positive nerve fibre content in the urothelium of the bladder was significantly increased in patients with hyperactivity and bladder pain syndrome, and capsaicin responses of rat dorsal root ganglion (DRG) neurons in the presence of N/OFQ were dose-dependently inhibited, indicating that activating OPRL1 represents a potential clinical pain management strategy^54^. However, studies by Seo indicated that curcumin and *Boswellia serrata* acted as nociceptin receptor antagonists to reduce pain by downregulating OPRL1 expression^55^. From the above results, the function of OPRL1 in modulating pain was complex and controversial, showing both analgesic and pain-promoting effects in different experimental settings, but it was undeniable that it had potential in pain treatment; however, before this step, we need to conduct more research to clarify the role of OPRL1.

CXCL13 was mainly expressed in secondary lymphoid tissues, such as lymph nodes, spleen and gut-associated lymphoid tissues. CXCL13 selectively attracts and guides B lymphocytes by acting on CXCR5 in lymphoid follicles^56^ and plays a role in the pathogenesis of autoimmune diseases, inflammatory diseases and tumours^57^. It was reported that proteoglycan biglycan induced CXCL13 expression and aggravated lupus nephritis in mice through TLR2/4 on macrophages and dendritic cells^58^. However, no similar finding was found in the intervertebral disc. However, the role of CXCL13 in pain was contrary to our expectation. CXCL13 and CXCR5 produce orofacial pain through ERK-mediated proinflammatory cytokines^59^. After ligation of the spinal cord nerve in mice, it was found that CXCL13 was upregulated in the spinal cord, and intrathecal injection of CXCL13 could cause hyperalgesia and activation of astrocytes^60^. This finding showed that the role of CXCL13 in intervertebral discs merits further study.

SST is an important regulator of the endocrine system with diverse effects and is widely distributed in the brain and periphery. Somatostatin inhibited the release of numerous secondary hormones by binding to high-affinity G-protein-coupled somatostatin receptors. Chapman reported that somatostatin has a marked antinociceptive function at the spinal level and a weaker inhibitory action at the peripheral nociceptor terminal but only in nociceptive states associated with peripheral inflammation^61^. Susan found that somatostatin and its receptors helped to inhibit cutaneous nociceptors^62^. Carlto^63^ and Heppelmann^64^ found that somatostatin not only worked as a peripheral analgesic agent but also served as a tonic control on peripheral nociceptors^65,66^. Huang found that pain sensitivity increased when eliminating somatostatin expression from primary afferent neurons, which indicated that somatostatin released from primary afferents was involved in inhibiting pain behaviour^67^. Thomas found that J-2156, a somatostatin receptor type 4 agonist, could relieve mechanical low back pain in a rat model^68^. However, surprisingly, the present study showed that somatostatin was lower in hypersamples, indicating that further research is warranted in this area, not only regarding the role of SST in nociception.

## Limitations

Despite rigorous bioinformatic analysis performed in this study, there were still some shortcomings in this work. Specifically, size of the dataset was small.

### Conclusion and future directions

Bioinformatic analysis could screen and identify significant gene biomarkers of low back pain caused by changes in the osmotic pressure of NP cells. The four genes (CCL5, OPRL1, SST, and CXCL13) were identified as significant gene biomarkers of low back pain. In particular, the expression of CCL5 and OPRL1 was most correlated with low back pain. Furthermore, this research could provide a reference for future in-depth research to identify gene biomarkers of low back pain.

## Methods

### Accessing the public dataset

The gene profile GSE1648 downloaded from the GEO database (https://www.ncbi.nlm.nih.gov/geo/) was produced by the [HG-U133A] Affymetrix Human Genome U133A Array (Platform GPL96). The gene profile consisted of 4 isotonic samples and 4 hypertonic samples. The samples were intervertebral disc NP cells obtained from patients undergoing discectomy prior to surgery for lumbar interbody fusion or lumbar disc herniation. The iso-osmotic media consisted of a defined cell culture medium (Ham’s F-12 with supplements as described above; 293 mOsm/kg H_2_O). The hyperosmotic media consisted of the same cell culture media supplemented with sucrose to a final osmolarity of 450 mOsm/kg H_2_O. The above data were obtained from L AWRENCE M’s cell culture^69^, which could be downloaded from the website (https://www.ncbi.nlm.nih.gov/geo/query/acc.cgi?acc=GSE1648).

### Identification of DEGs

The limma (Linear Models for Microarray Analysis) R package^70^, which could perform the T test, emerged as one of the most widely used statistical tests for identifying differentially expressed genes. The package could be obtained from the open website (http://www.bioconductor.org/packages/release/bioc/html/limma.html). As a fully functional package, the limma R package included the original data input and preprocessing capabilities of complementary DNA (cDNA) chips extracted from NP cells, as well as a linear model for analysing differentially expressed genes. We screened DEGs between isotonic and hypertonic NP cells by utilizing the limma package with an adjusted P-value < 0.05 and a log (fold change) >1 or log (fold change) <−1 as the cut-off criteria. The volcano plot was drawn by R language.

### Functional annotation for DEGs with database for annotation, visualization and integrated discovery (DAVID)

The DAVID (https://david.ncifcrf.gov/home.jsp) (version 6.8)^71^ was one online analysis tool suite with the functional annotation for Gene Ontology^72^ and Kyoto Encyclopedia of Genes and Genomes (KEGG)^73^. To perform the Gene Ontology and KEGG analysis of DEGs, the DAVID online tool was implemented. First, we clicked on the “Functional Annotation” on the website (https://david.ncifcrf.gov/home.jsp). Second, we entered the gene list, selected identifiers as official gene symbols, selected list types as gene lists, and submitted lists. Third, we selected to limit annotations and background by Homo species. Finally, the enrichment results of Gene Ontology and KEGG are presented. P-values < 0.05 indicated significance.

### Pathway and process enrichment analysis with metascape analysis

Furthermore, pathway and process enrichment analyses were performed by Metascape (http://metascape.org/gp/index.html#/main/step1)^74^. For given DEGs identified between the isotonic NP cells and hypertonic NP cells by the above analysis (see the “Identification of DEGs” section), function and pathway enrichment analysis was carried out with the following ontology sources: Gene Ontology and KEGG Pathway. Terms with a P < 0.01, a minimum count of 3, and an enrichment factor >1.5 were collected and grouped into clusters.

### Enrichment analysis of gene ontology and KEGG by gene set enrichment analysis (GSEA)

The gene sequences were obtained from isotonic and hypertonic NP cells. GSEA was able to analyse all gene sequences of the samples from different groups and export them into two groups in the form of a gene expression matrix, and all genes were sequenced first and then used to indicate the trend of gene expression level between the two groups^75^. In this study, we performed GESA analysis on gene sequences of nucleus pulposus under isotonic and hypertonic NP cells as follows. GESA software analysed and sorted genes according to the algorithm after importing gene annotation files, reference function sets and gene data of nucleus pulposus under isotonic and hypertonic conditions, and then we obtained a gene sequence table. After that, GESA software analysed the positions of all genes and accumulated them to obtain enrichment scores.

### Construction and analysis of the protein-protein interaction (PPI) network, significant module, and hub gene network

First, Metascape (http://metascape.org/gp/index.html#/main/step1)^74^ was used to construct the PPI network and screen the significant module.

Moreover, the Search Tool for the Retrieval of Interacting Genes (STRING, http://string.embl.de/) was also applied to construct the PPI network, and Cytoscape was used to present the network^73^. Cytoscape (version 3.6.1) was a free visualization software^76^. The Molecular Complex Detection tool (MCODE) (version 1.5.1)^77^ could screen and identify the most significant module in the PPI network. When the degrees were set (degrees >10), the hub genes were excavated.

### Identification and analysis of significant genes

A Venn diagram was delineated to identify significant common genes among “Metascape_MCODE”, “Cytoscape_MCODE”, and “Cytoscape_cytoHubba” by FunRich software (http://www.funrich.org). Summaries for the function of 4 significant genes were obtained via GeneCards (https://www.genecards.org/). The R language was used to perform the clustering analysis of significant genes based on the gene expression level. Pearson’s correlation test was performed to complete the correlation analysis among the hub genes.

### Identification of significant genes associated with pain and intervertebral disc degeneration

The comparative toxicogenomics database (http://ctdbase.org/) is a web-based tool that provides information about interactions between chemicals and gene products and their relationships to diseases^78^. The relationships between the significant genes and “pain and intervertebral disc degeneration” were analysed via the tool.

### Cell isolation and culture

Human IVD tissue was obtained from patients (60±5 years old) undergoing discectomy prior to surgery for lumbar disc herniation (n = 50) or lumbar interbody fusion (n = 70). Before the surgery, NRS was obtained from all the patients. The research conformed to the Declaration of Helsinki and was authorized by the Human Ethics and Research Ethics Committees of Beijing Ditan Hospital. Informed consent was obtained from all patients. All tissue was harvested from the central NP region. Sequential pronase–collagenase digestion was used to isolate NP cells. The osmotic pressures between inside and outside of the NP cells were measured by an osmometer [VAPRO 5600 (5520), New York, US]. The NP cells were cultured in RPMI-1640 (GIBCO, Gran Island, NY, USA) containing 1% penicillin-streptomycin and 10% foetal bovine serum (FBS; HyClone, Logan, UT, USA) at 37 °C with 5% CO_2_. Cell culture was performed under normoxia. Cell culture medium was exchanged for either hyperosmotic (450 mOsm/kg H_2_O) or iso-osmotic media (293 mOsm/kg H_2_O) after 24 h^69^. The osmolarity was altered by using both sucrose and NaCl^17^.

### Quantitative real-time PCR

The relative mRNA expression levels of CCL5, OPRL1, CXCL13, and SST were measured by quantitative real-time PCR. Total RNA extraction, cDNA synthesis, and qPCR were performed. Total mRNA was extracted from NP cells with TRIzol reagent (Invitrogen, Beijing, China) according to the manufacturer’s protocol. One microgram of total RNA was used to generate first strand cDNA using random primers and SuperScript II reverse transcriptase (Invitrogen). Real-time PCR was performed in triplicate using the SYBR PrimeScript RT-PCR Kit (Takara, Dalian). The expression of GAPDH was measured as an internal control. Thermocycler conditions included an initial hold at 50 °C for 2 minutes and then 95 °C for 10 minutes followed by a two-step PCR programme of 95 °C for 15 seconds and 60 °C for 60 seconds repeated for 40 cycles on an Mx4000 system (Applied Biosystems, Foster City, CA), on which data were collected and quantitatively analysed^79^. The expression level of mRNA was demonstrated as the fold change relative to the control group^80^. The primer sequences used in the qPCR are shown in Table 9.

**Table 9:** Primers and their sequences for PCR analysis.

Primer	Sequence (5′–3′)
CCL5-hF	GGAGAATCGCTTGAACCC
CCL5-hR	GGGTTCAAGCGATTCTCC
OPRL1-hF	GTTCTGTGAGTCCCTGTCTGTG
OPRL1-hR	CCAGCCTACCTGAGGATGAC
SST-hF	CCGTATGTTTGAGATTGTG
SST-hR	GACCGTTCTGGTAAGATAAA
CXCL13-hF	TAGGGCTAAAGGGTTGTT
CXCL13-hR	TGATGAGTTGATGGGTGC

### Identification of the miRNA-gene pairs of the significant genes

TargetScan (www.targetscan.org) is a web-based database that can predict biological targets of miRNAs. In our study, the miRNA-gene pairs of the significant genes were screened out with TargetScan.

### Statistical analysis

The data are expressed as the percentage of total and mean ± SD. When two groups were compared, Student’s t-test was used to determine statistical significance. Double Delta Ct Analysis was used for the PCR statistics.

By using Pearson’s correlation test, associations between the NRS, osmotic pressure, and the expression of CCL5 and OPRL1 were analysed. We used linear regression analysis to explore the linear correlations between NRS, osmotic pressure, and the expression of CCL5 and OPRL1. The Spearman-rho test was executed to compare NRS, osmotic pressure, and the expression of CCL5, SST, CXCL13, and OPRL1 for the correlation analysis. Univariate linear regression analysis between NRS and relevant gene expression was performed. When any analytic results reached a liberal statistical threshold of p < 0.2 for each comparison, the risk factors were forced into the multivariable linear regression model to confirm independent risk factors for the NRS. Variance inflation factors were calculated to quantify the severity of multicollinearity. To identify the residual distribution, a histogram and Shapiro-Wilk test were conducted, and we concluded that residuals accurately modelled the normal distribution. Univariate and multivariate logistic regression analysis was used to calculate the odds ratios (ORs) of each variable for NRS. A receiver operating characteristic curve analysis was performed to determine the ability of the expression of CCL5 and OPRL1 to predict the NRS.

All statistical analyses were conducted using SPSS software, version 23.0 (IBM Corp., Armonk, NY, USA). A p-value < 0.05 was considered to be significant^81^.

### Construction of neural network modelling and cubic spline interpolation between the expression of CCL5 and OPRL1 and NRS

The training group was divided into training data and calibration data randomly according to the proportion of 7:3. There were 77 individuals in the training data and 33 individuals in the calibration data. Furthermore, a total of 10 individuals were used as the validation data. MATLAB (version: 8.3, NY, US) was used to accomplish the normalization processing of variable values, network initialization, network training, and network simulation. The number of input neurons in the input layer was the same as the number of input variables, and the number was two. The hidden layer was designed as 1 layer, and the output layer was also designed for 1 layer. One output variable was NRS. Then, the forecast model was established with a hidden unit number of 6. When training to 2000 steps after repeated training, the falling gradient is 0, and the training speed is uniform. At the same time, the training error was 0.0076566, and the R (relativity) value reached 0.98987. Cubic spline interpolation was performed to predict the high-risk warning range of NRS by using the expression of CCL5 and OPRL1^82^.

### Ethics approval and consent to participate

The data of this research were downloaded from the GEO database public website. The research conformed to the Declaration of Helsinki and was authorized by the Human Ethics and Research Ethics Committees of Beijing Ditan Hospital. All institutional and national guidelines for the care and use of participants were followed.

## Data Availability

The datasets used and/or analysed during the current study are available from the corresponding author on reasonable request.
